# Nanoprecipitation of Biocompatible Poly(malic acid) Derivative, Its Ability to Encapsulate a Molecular Photothermal Agent and Photothermal Properties of the Resulting Nanoparticles

**DOI:** 10.3390/molecules26247703

**Published:** 2021-12-20

**Authors:** Marian Gabriela Vargas Guerrero, Jean-Baptiste Pluta, Nathalie Bellec, Sandrine Cammas-Marion, Franck Camerel

**Affiliations:** 1 Institut des Sciences Chimiques de Rennes (ISCR), Ecole Nationale Supérieure de Chimie de Rennes, CNRS, University of Rennes, UMR 6226, F-35042 Rennes, France; marian-gabriela.vargasguerrero@etudiant.univ-rennes1.fr (M.G.V.G.); jean-baptiste.pluta@univ-rennes1.fr (J.-B.P.); nathalie.bellec@univ-rennes1.fr (N.B.); 2 Institut NUMECAN (Nutrition Metabolisms and Cancer), INSERM, INRAE, University of Rennes, UMR_A 1341, UMR_S 1241, F-35000 Rennes, France

**Keywords:** biocompatible nanoparticles, poly(benzyl malate), nanoprecipitation, metal-bis(dithiolene), photothermal

## Abstract

Biocompatible nanoparticles (NPs) of hydrophobic poly(benzyl malate) (PMLABe) were prepared by nanoprecipitation. The influence of nanoprecipitation parameters (initial PMLABe, addition rate, organic solvent/water ratio and stirring speed) were studied to optimize the resulting formulations in terms of hydrodynamic diameter (Dh) and dispersity (PDI). PMLABe NPs with a Dh of 160 nm and a PDI of 0.11 were isolated using the optimized nanoprecipitation conditions. A hydrophobic near infra-red (NIR) photothermally active nickel-bis(dithiolene) complex (Ni8C12) was then encapsulated into PMLABe NPs using the optimized nanoprecipitation conditions. The size and encapsulation efficiency of the NPs were measured, revealing that up to 50 weight percent (wt%) of Ni8C12 complex can efficiently be encapsulated with a slight increase in Dh of the corresponding Ni8C12-loaded NPs. Moreover, we have shown that NP encapsulating Ni8C12 were stable under storage conditions (4 °C) for at least 10 days. Finally, the photothermal properties of Ni8C12-loaded NPs were evaluated and a high photothermal efficiency (62.7 ± 6.0%) waswas measured with NPs incorporating 10 wt% of the Ni8C12 complex.

## 1. Introduction

**Figure 1:**
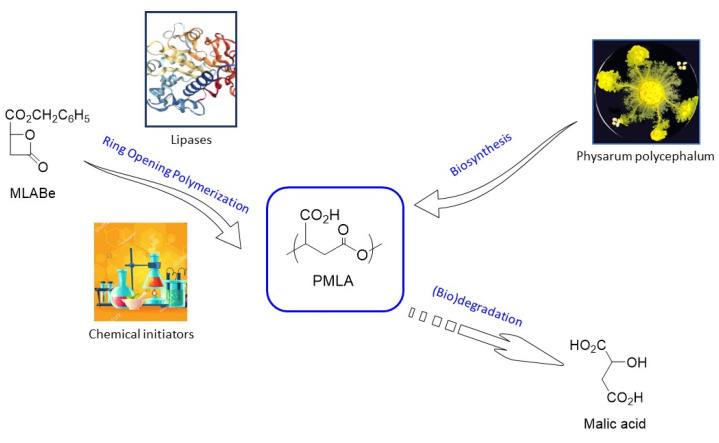
Structure of PMLA, methods of (bio)synthesis and degradation product.

Since the “magic bullet” postulated by Paul Ehrlich at the beginning of the 20th century [1], tremendous progress has been realized in the field of nanomedicine [2], as highlighted by the increases number of nanocarriers already approved or under clinical trials [3,4,5]. It should be noted that nanocarriers designed for use in humans have to answer to very strict specifications. Indeed, from their administration to their total elimination, nanocarriers and their constituents, have to: (i) be biodegradable or bioassimilable and biocompatible; and (ii) have a low opsonin recognition, a high loading capacity and a controlled drug release at the site of action. Among the nanovectors developed as drug carriers, polymeric nanoparticles (NPs) have attracted a great deal of attention because of the numerous possibilities of polymeric material modification allowing their physico-chemical properties and those of corresponding nanocarriers to be adjusted to improve drug loading capacity and overall efficiency [5,6,7,8,9]. To design biocompatible and biodegradable nanocarriers, various natural and/or synthetic polymers have been used, among which the best known and most widely used are poly(lactic acid) and its derivatives [3]. However, these polyesters lack functional groups useful for further modifications. In this context, poly(malic acid) (PMLA, Figure 1), developed specifically for biomedical applications, is of interest because: (i) it can be easily modified to introduce molecules of interest thanks to its lateral carboxylic acid functions, (ii) it is known to be biodegradable into malic acid, a Kreps’ cycle molecule, and (iii) it can be either biosynthesized [7,10] or synthesized through ring opening polymerization (ROP) of β-substituted β-lactones using either lipases or carboxylates as initiators [11,12,13].

The encapsulation of photothermal agents into NPs is under investigation to develop stimuli-responsive NPs for diagnostic, photothermal therapy or photo-controlled drug release [14,15]. Light, as a stimulus, can enable good spatio-temporal activation for localized diagnostic and treatment. Several types of molecular photothermal agents, such as cyanine, Bodipy, croconaine and porphyrin derivatives have been encapsulated into organic NPs to develop photoactive systems in the NIR region [16,17,18,19,20]. However, their uses are usually limited due to their instability and their photobleaching upon laser irradiation [21].

Metal-bis(dithiolene) complexes are photothermally and photochemically stable, strong NIR absorbers with efficient photothermal properties under NIR irradiation in solid–state, in gel and in solution [22]. Recently, it has also been demonstrated that such hydrophobic metal-bis(dithiolene) complexes can be encapsulated into the hydrophobic inner-core of NPs based on PEG-*b*-PMLABe block copolymers for the controlled release of doxorubicin under NIR laser irradiation [23].

In our quest to develop biocompatible NPs encapsulating metal-bis(ditiolene) phothermally-active complexes for in vivo theranostic applications, we now turn on our attention on the hydrophobic PMLABe polymer. Biocompatible PMLABe-based NPs are easily and reproducibly obtained using the simple nanoprecipitation method, first described by Thioune et al. [24], which consisted of the rapid addition of hydrophobic polymer or amphiphilic block copolymer, solubilized into a water-miscible organic solvent, into an aqueous medium that may or may not contain surfactants. PMLABe was shown to self-assemble into aqueous medium without the presence of a surfactant, thus leading to stable well-defined NPs [25]. Nevertheless, the influence of key nanoprecipitation parameters such as stirring speed, addition rate, initial concentration of PMLABe into organic solvent and the organic solvent/water ratio on NP characteristics (hydrodynamic diameters and dispersity) was never evaluated. Moreover, the influence of initial number of hydrophobic molecules to be encapsulated on the characteristics and encapsulation efficiency of NPs has never been studied.

Consequently, in the present work, first, the influence of the previously cited parameters on the main characteristics (hydrodynamic diameter, dispersity and hydrophobic photothermal complex encapsulation efficiency) of PMLABe_73_-based NPs were evaluated in order to select the best conditions leading to NPs with the lowest hydrodynamic diameter (Dh) and dispersity (PDI). Second, after selecting the best nanoprecipitation parameters, the encapsulation of a hydrophobic photothermal nickel-bis(dithiolene) Ni8C12 was investigated to determine the highest encapsulation efficiency (E.E.). Finally, the photothermal properties of the PMLABe_73_ NPs incorporating the dithiolene complex were evaluated under NIR laser irradiation in aqueous solutions.

## 2. Results and Discussion

**Scheme 1:**
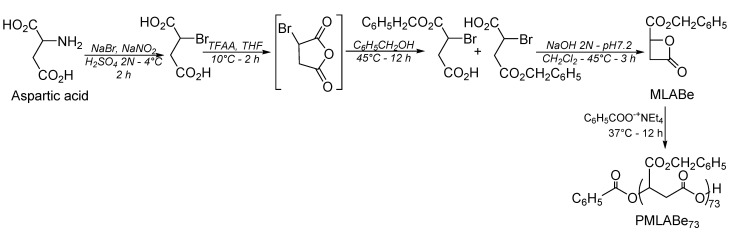
Synthetic route to PMLABe_73_.

For the present study, the selected poly(benzyl malate), PMLABe_73_, was synthesized through the anionic ring opening polymerization (aROP) of benzyl malolactonate (MLABe), a monomer prepared in four steps starting from DL-aspartic acid as described previously, in the presence tetraethylammonium benzoate as an initiator (Scheme 1) [25].

The molecular structure and the weight average molar mass were ascertained by ^1^H NMR spectroscopy and size exclusion chromatography (SEC) (Figures S1–S3). The theoretical molar mass of PMLABe_73_ was fixed by the monomer (MLABe)/initiator (tetraethylammonium benzoate) ratio and chosen at 15,000 g/mol (number of repeating benzyl malate units: 73). The polymerization reaction was followed by FT-IR, and was stopped when all the monomer was consumed as highlighted by the disappearance of the lactonic band at 1850 cm^−1^ (Figure S1). The purified PMLABe_73_ had good structure as highlighted by its ^1^H NMR spectrum (Figure S2), and a measured weight average molar mass close to the theoretical one (measured Mw was lower than the theoretical one because of sample adsorption onto the SEC column and the different nature of the standards used) with a dispersity showing a homogenous polymer sample (Figure S3).

This hydrophobic polymer was then used to set up the optimal nanoprecipitation leading to well-defined, reproducible NPs.

### 2.1. Influence of the Principal Nanoprecipitation Conditions onto NPs Characteristics

The influence of four nanoprecipitation key parameters (stirring speed, addition rate, initial concentration of PMLABe into organic solvent and the organic solvent/water ratio) on the hydrodynamic diameter and dispersity of the pure PMLABe_73_-based NPs was evaluated.

**Figure 2:**
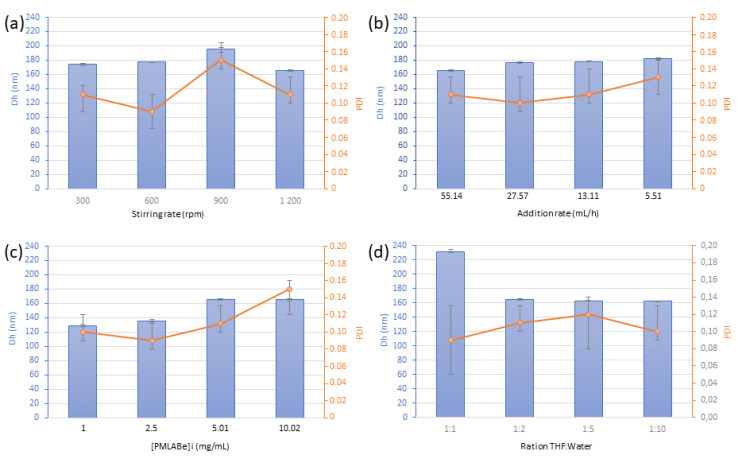
Evolution of NP hydrodynamic diameter (Dh) and dispersity (PDI), measured by DLS, as a function of: (**a**) the stirring rate during nanoprecipitation, using a concentration of polymer of 5 mg/mL, an addition rate of 55.14 mL/h and a THF:water ratio of 1:2; (**b**) the organic phase addition rate, using a concentration of polymer of 5 mg/mL, a stirring speed of 1200 rpm and a THF:water ratio of 1:2; (**c**) the PMLABe_73_ initial concentration, using a stirring speed of 1200 rpm, an addition rate of 55.14 mL/h and a THF:water ratio of 1:2 and (**d**) the THF:water ratio, using a concentration of polymer of 5 mg/mL, a stirring speed of 1200 rpm and an addition rate of 55.14 mL/h.

First, NP diameters and dispersity were measured by dynamic light scattering (DLS) on an NP suspensions obtained by nanoprecipitation of PMLABe_73_ as a function of the stirring rate. For this purpose, PMLABe_73_ solubilized into THF at a concentration of 5 mg/mL was added into water using a ratio of THF:water of 1:2 and an addition rate of 55.14 mL/h. The stirring speed was varied from 300 to 1200 rpm (round per minute), and the NP suspensions thus obtained were analyzed by DLS (Figure 2a).

As shown in Figure 2a, the hydrodynamic diameter of the NPs was slightly influenced by the stirring rate applied during the nanoprecipitation, ranging from 160 to 200 nm, with the lowest value being obtained at a stirring rate of 1200 rpm. The dispersity of the NP suspensions varied from 0.07 to 0.17, with the lowest PDI obtained at a stirring rate of 600 rpm.

The stirring speed weakly affected the nanoparticle size. PDI, however, appears to be more sensitive to the stirring speed. A high stirring speed results in the smallest NPs with good PDI.

Subsequently, a stirring speed of 1200 rpm during the nanoprecipitation step was chosen and the addition rate was varied between 55.14 mL/h, a very fast organic solution of PMLABe_73_ addition into water, and 5.51 mL/h, a very slow organic solution of PMLABe_73_ addition into water (Figure 2b). As shown in Figure 2b, the NP hydrodynamic diameter and dispersity values slightly increased with the decrease of the addition rate. The NP diameter ranged from 160 nm at high addition speed to 180 nm at low addition speed, and the PDI increased from 0.07 to 0.14. The lowest hydrodynamic diameters and dispersity values were thus obtained from a very fast addition of the organic phase containing the PMLABe_73_ in the aqueous phase. The addition rate of 55.14 mL/h was thus selected for the rest of the present study.

An important parameter that might have a great influence on both the hydrodynamic diameter and dispersity of NP suspensions is the initial hydrophobic polymer concentration in the organic solvent. Indeed, a too high polymer concentration in the organic solvent and therefore in the aqueous phase might lead to the formation of aggregates, or even a precipitate, while a too low polymer concentration in both the organic and aqueous phases might lead to unstable nanoaggregates or only unimers in suspension into the aqueous phase (Figure 2c). As shown by results shown in Figure 2c, both the hydrodynamic diameter and dispersity slightly increased when the initial concentration of PMLABe_73_ varied from 1 to 10 mg/mL. However, it can be noticed that the size increase was very limited, between 1 and 5 mg/mL.

Thus, the initial PMLABe_73_ concentration of 5 mg/mL in THF was kept to evaluate the influence of the THF:water ratio on the NP characteristics (Figure 2d). As shown by the results shown in Figure 2d, the THF:water ratio had a significant influence on the two studied parameters. For the same initial PMLABe_73_ concentration, the hydrodynamic diameter of the NPs decreased from 220 nm to 160 nm with an increase in the THF:water ratio, while the dispersity of the resulting NP suspensions varied from 0.05 to 0.13. From these results, it appeared that THF:water ratios of 1:2 and 1:5 might obtain NP suspensions with acceptable hydrodynamic diameter and dispersity lower than 0.11 (Figure 2d).

Based on these experiments, it appears that the use of a stirring rate of 1200 rpm, an addition speed of 55.14 mL/h, a PMLABe_73_ concentration of 5 mg/mL in THF and a THF:water ratio lower than 1:2 is suitable to obtain good quality nanoparticle dispersion with a hydrodynamic diameter around 160 nm and a low dispersity of around 0.11. Thus, these conditions were kept to study the influence of the initial quantity of hydrophobic molecules to be encapsulated on the characteristics and encapsulation efficiency of the NPs.

**Figure 3:**
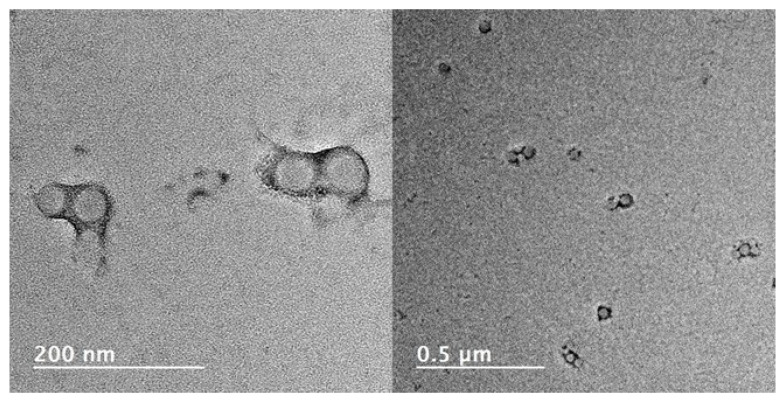
TEM images of the PMLABe_73_ NPs obtained at a stirring rate of 1200 rpm, a polymer concentration of 5 mg/mL, an addition rate of 55.14 mL/h and a THF:water ratio of 1:2.

The PMLABe_73_ NPs obtained using the optimized nanoprecipitation conditions detailed above were further analyzed by transmission electron microscopy (TEM) and the images show that the nanoparticles had a spherical shape (Figure 3). The images also confirm a low dispersity, with a mean diameter of around 46 ± 14 nm. The average diameter measured by TEM was lower than that measured by DLS because the hydration layer is not visible by TEM.

### 2.2. Evaluation of the Effect of Initial Amount of Photothermal Agent on NPs Characteristics and Encapsulation Efficiency

**Figure 4:**
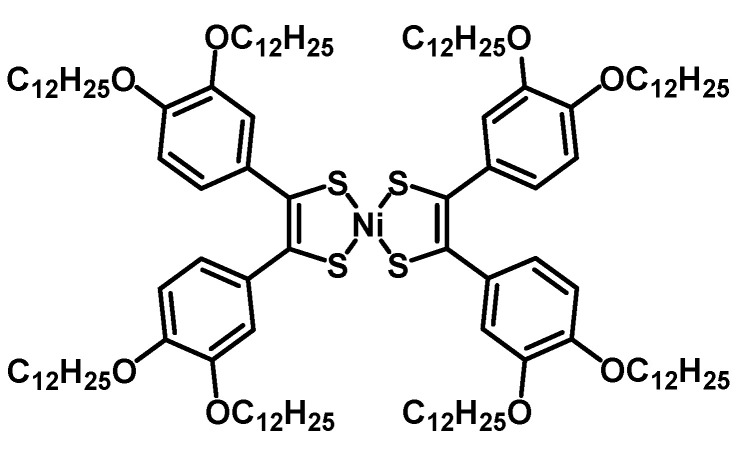
Structure of the selected photothermal agent Ni8C12.

Because these NP suspensions were first designed to allow the vectorization of hydrophobic photothermal agents to improve site-specific PhotoThermal therapy (PTT), the nickel-bis(dithiolene) complex Ni8C12 (Figure 4) was selected to evaluated the effect of initial quantity of this photothermal agent on NP characteristics.

Under laser irradiation in the NIR region Ni8C12 is a highly stable hydrophobic photothermal agent. The photothermal properties of this metal complex were deeply investigated in solution and it has a photothermal conversion efficiency of 48% under 940 nm laser irradiation in toluene [26].

**Figure 5:**
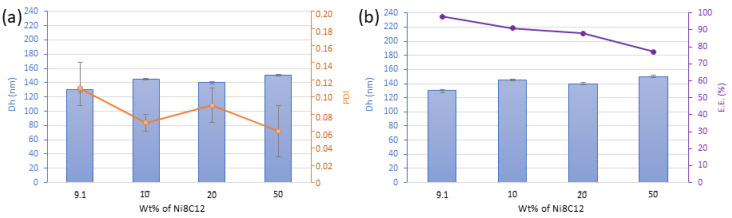
(**a**) Evolution of NPs hydrodynamic diameter (Dh) and dispersity (PDI) measured by DLS, as a function weight percent of Ni8C12, and (**b**) evolution of NP hydrodynamic diameter (Dh) and encapsulation efficiency (E.E.) as a function weight percent of Ni8C12 (for E.E., only one measurement was performed).

Several PMLABe_73_ solutions at 5 mg/mL in THF with percentages in weight of the photothermal complex Ni8C12 from 9.1 weight percent (wt%) to 50 wt% were prepared. These organic solutions were further nanoprecipitated into water at addition rate of 55.14 mL/h and a stirring speed of 1200 rpm. The evolution of the hydrodynamic diameter, dispersity and encapsulation efficiency (E.E.) was followed by DLS (Dh and PDI) and UV analysis (E.E.). Figure 5 shows the obtained results.

As shown in Figure 5a, no significant differences were observed between NP suspensions loading the photothermal agent Ni8C12, no matter the weight percentage selected. However, the encapsulation efficiency (E.E.) decreased with the increase in the initial quantity of Ni8C12 (Figure 5b).

**Figure 6:**
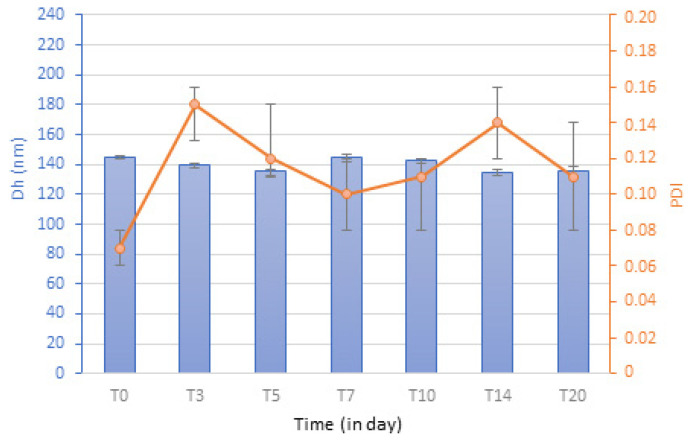
Evolution of Dh and PDI of Ni8C12-loaded PMLABe73-based NPs with storage time at 4 °C.

The stability of the Ni8C12-loaded NPs was evaluated under storage conditions (4 °C). NPs loaded with 10 wt% of Ni8C12 were prepared as described above, and their stability was followed by measuring the evolution of their hydrodynamic diameter and dispersity (Figure 6).

As shown by data gathered in Figure 6, the hydrodynamic diameter of the Ni8C12-loaded NPs was stable under storage at 4 °C, while the dispersity fluctuated during the first 5 days. After th equilibration period of 5 days, no significant evolution of hydrodynamic diameter and dispersity was noted on the NP suspensions stored at 4 °C.

These results show that large amounts of hydrophobic Ni8C12 complex can be encapsulated inside the NPs without affecting their diameter and polydispersity PMLABe is a good biocompatible polymer to form high quality NP suspensions incorporating a large amount of hydrophobic photothermal agents, showing a long stability at 4 °C compatible with in vivo applications.

### 2.3. Photothermal Properties of PMLABe-Based NPs Encapsulating the Photothermal Agent

To investigate the photothermal properties of these PMLABe-based NPs encapsulating photothermal nickel-bis(dithiolene) complexes, we selected the NPs with one of the highest encapsulation efficiencies (E.E.) and the lowest PDI, i.e., the NPs containing 10 wt% Ni8C12. In addition, NPs with low complex content were used to demonstrate the high photo-thermal efficiency of this class of molecular agents. The absorption spectrum of an aqueous suspension of PMLABe NPs incorporating 10 wt% of Ni8C12 complex is presented in Figure S4. A strong absorption band centered at 901 nm, extending from 740 nm up to 1200 nm was observed, and it confirms the encapsulation of the nickel-bis(dithiolene) complex into the PMLABe NPs. In fact, this low-energy absorption band is characteristic of neutral Ni-bis(dithiolene) complexes and is attributed to an electronic transition from the HOMO (Lπ) of b_1u_ symmetry to the LUMO (Lπ *-d_xy_) with a metallic character of b_2g_ symmetry [27]. The absorption band observed in the UV region is assigned to π–π* transitions localized at the dithiolene ligands.

**Figure 7:**
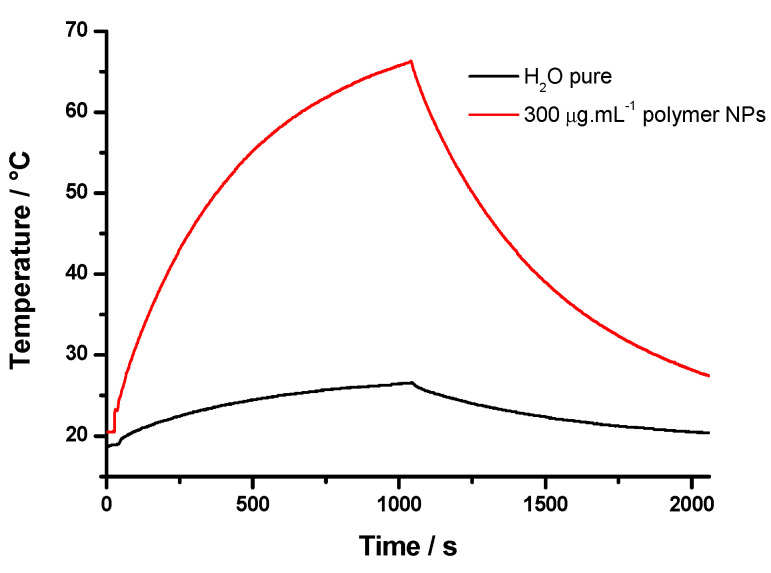
Temperature profile recorded on a suspension of PMLABe NPs incorporating 10 wt% of Ni8C12 complexes in water (C_pol_ = 300 µg·mL^−1^) under 880 nm laser irradiation (17 min irradiation 3 W·cm^2^ + 17 min laser OFF).

The photothermal properties of the resulting PMLABe NPs incorporating 10 wt% Ni8C12 complex were evaluated by irradiating aqueous suspensions at different concentrations with a continuous 880 nm laser with adjustable laser power from 1 to 3 W·cm^−2^. This laser wavelength was selected for its close proximity to the absorption maximum of the encapsulated complexes. The solutions were irradiated for 17 min (1020 s) to reach a steady state. After turning off of the laser, the cooling regimes of the solutions were recorded for another 17 min. Figure 7 shows a typical recorded temperature profile. The recorded temperature increase (ΔT) of a solution of Ni8C12 loaded PMLABe NPs at a concentration of 300 µg·mL^−1^ under 880 nm laser irradiation and a laser power of 3 W·cm^2^ was 45.6 °C, whereas the ΔT was only 7.6 °C with pure water, highlighting a clear photothermal effect.

**Figure 8:**
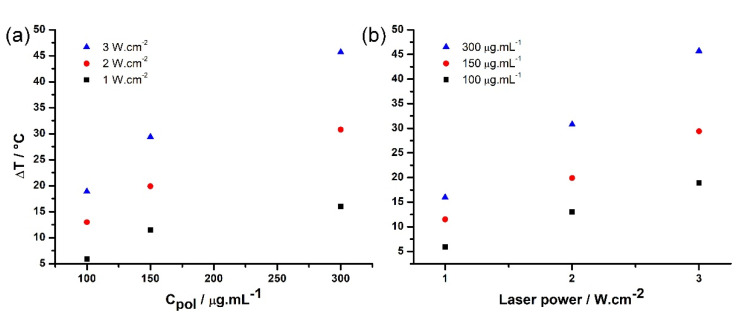
Temperature increase (ΔT) recorded as a function of the concentration of polymer (**a**) and of the laser power (**b**) (each point corresponds to a single measurement).

The temperature increases were also recorded as a function of the laser power and the concentration of NPs (Figure 8). The ΔT increased linearly with the concentration of polymers, i.e., the concentration of the nickel-bis(dithiolene) complex, and the laser power, reflecting that the temperature increase can be finely tuned by a proper choice of the laser power and the concentration of the doped polymer.

The photothermal efficiency (η) value of the photothermal agent was calculated according to the following equation described by Roper and al. based on the energy balance of the system [28]:

η = (hSΔT_max_ − Q_water_)/I × (1 − 10^−A^) (1)

where h is the heat-transfer coefficient, S is the surface area of the container, ΔT_max_ is the maximum steady-state temperature change of the solution, I is the power of the laser and A the absorbance at 808 or 940 nm. Q_water_ was measured independently and represents heat dissipated from light absorbed with a pure water solution. The hS value is derived according to Equation (2):

τs = m_water_C_water_/hS (2)

where τs is the sample system time constant, m_water_ (2 g) and C_water_ (4.18 J·g^−1^·K^−1^) are the mass and the heat capacity of water. τs is given by the slope of the linear fitting from the time of the laser off state vs. –ln(ΔT/ΔTmax).

A photothermal efficiency (η) of 62.7 ± 6.0% (95% confidence interval, N = 9, σ_8_ = 7.9 %, t_95%;_ σ_8_ = 2.306) was determined in the PMLABe NPs incorporating 10 wt% of Ni8C12 complex suspensions. It should be noted that the photothermal efficiency measurements must be carried out on sufficiently diluted solutions to avoid colloidal diffusion which can lead to an underestimation of the photothermal activity.

**Figure 9:**
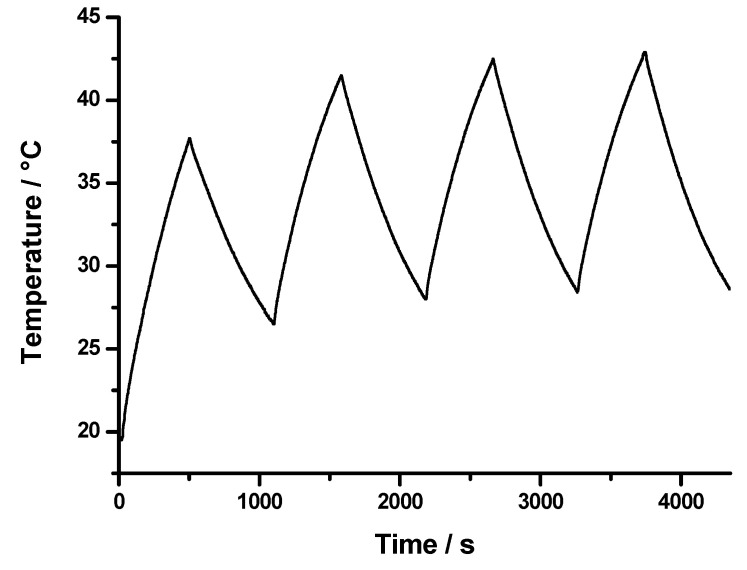
Stability measurement on a suspension of PMLABe NPs incorporating 10 wt% of Ni8C12 complex (C_pol_ = 300 µg·mL^−1^) over three heating cycles under 880 nm laser irradiation (3 W·cm^−2^).

Finally, the stability of the photothermal NPs under laser irradiation was evaluated by successive heating and cooling cycles. The suspensions were irradiated for 8 min, instead of the 17 min previously used for the η measurements, explaining the lower maximal temperature reached. The NPs appear to be highly stable after several heating and cooling cycles since no fatigue in the temperature increase was observed (Figure 9). The increase of the maximal temperature observed between each cycle was attributed to a slower cooling speed than the heating speed. DLS measurements also confirm that the mean hydrodynamic diameter of the photothermal NPs was weakly affected by prolonged laser irradiation. The average diameter of 123 nm (PDI 0.11) measured after laser irradiation was close to that of the initial suspension (140 nm, PDI 0.12).

## 3. Materials and Methods

A PMLABe_73_ polymer was synthesized as previously described [26]. Briefly, 1.4 mg of tetraethyl ammonium benzoate (initiator) was first dried under vacuum overnight in order to eliminate any trace of water. A total of 1.10 g of benzyl malolactonate (MLABe) was transferred using a cannula to the flask containing the initiator under a nitrogen atmosphere and the temperature was set to 37 °C. The completion of the reaction was monitored by FT-IR analysis (disappearance of the C=O lactonic band at 1850 cm^−1^, Figure S1). The polymer was then dissolved in 1 mL of acetone and added dropwise into 200 mL of ethanol under constant stirring. After the precipitation of the polymer, the supernatant was removed and the recovered polymer was placed under vacuum overnight to remove all traces of solvent. The weight average molar mass (Mw) and the dispersity (Đ = Mw/Mn) values of the PMLABe_73_ were measured by size exclusion chromatography (SEC) in THF at 40 °C (flow rate = 1.0 mL/min) on a GPC2502 Viscotek apparatus equipped with a Viscotek VE 3580 RI refractive index detector, a Viscotek TGuard guard column, Org 10 mm × 4.6 mm, an LT5000 L gel column (for samples soluble in organic medium) 300 mm × 7.8 mm, and GPC/SEC OmniSEC software. The polymer samples were dissolved in THF (2 mg/mL). All elution curves were calibrated with polystyrene standards. PMLABe_73_ was also characterized by ^1^H NMR (400 MHz, Acetone-d_6_), δ (ppm): 2.97 (m, 2nH); 5.14 (m, 1nH); 5.53 (m, 2nH); 7.33 (m, 5nH); 8.04–8.06 (d, 2H) (Figure S2). SEC (THF, 40°C, polystyrene standards): Mw = 9500 g/mol, Ð = 1.51 (Figure S3).

The nickel-bis(dithiolene) Ni8C12 complexes were prepared as previously reported [29]. Briefly, 3,4,3′,4′-tetra(dodecanoxy)benzyl was sulfureted with P_4_S_10_ in dioxane, followed by hydrolysis of the intermediate phosphorous thioesters in the presence of a nickel salt such as NiCl_2_·6H_2_O to directly afford the oxidized, neutral nickel complex. The green compound was purified by column chromatography on silica gel and crystallized by slow evaporation of dichloromethane from a CH_2_Cl_2_/MeOH mixture. ^1^H NMR (300 MHz, CDCl_3_) δ 7.57 (d, J = 2.0 Hz, 4H), 7.43 (dd, J = 8.4, 2.0 Hz, 4H), 6.85 (d, J = 8.5 Hz, 4H), 4.16–3.90 (m, 16H), 1.96–1.72 (m, 16H), 1.64–1.13 (m, 144H), 0.88 (t, J = 6.5 Hz, 24H).

The molar absorptivity (ε) of Ni8C12 in THF at 940 nm was calculated using the Beer–Lambert law by measuring the average absorbance at 940 nm of three solutions of Ni8C12 in THF (C = 2 × 10^−5^ mol·L^−1^).

Ultrapure water (18.2 Mohm) was produced on a Purelab Classic ELGA purification system and NORMAPUR THF was purchased from VWR Chemicals and used as received. A total of 300 (^1^H) MHz NMR spectra were recorded on a Bruker Avance 300 spectrometer at room temperature using perdeuterated solvents as internal standards. FT-IR spectra were recorded on an Avatar 320FT-IR Thermo Nicolet spectrometer between 500 and 4000 cm^−1^ by direct measurement.

PMLABe_73_-based NPs were prepared using the nanoprecipitation method described by Thioune et al. based on the self-assembly of a hydrophobic polymer in aqueous medium [25]. To this end, the polymer was first solubilized in THF, a water-miscible organic solvent, and then rapidly added to an aqueous solution. As initial conditions, the PMLABe_73_ (5 mg) was thus solubilized in 1 mL of THF and then rapidly added to 2 mL of ultrapure water under vigorous stirring (1200 rpm) with an addition rate of 55.14 mL/h. The mixture was then stirred at room temperature for 10 min. In this process, the hydrophobic polymer (PMLABe_73_) aggregated in contact with the water, thus leading to the formation of NPs. The THF was then evaporated under reduced pressure (rotary evaporator) and the final volume was adjusted to 2 mL by the addition of ultrapure water.

**Scheme 2:**
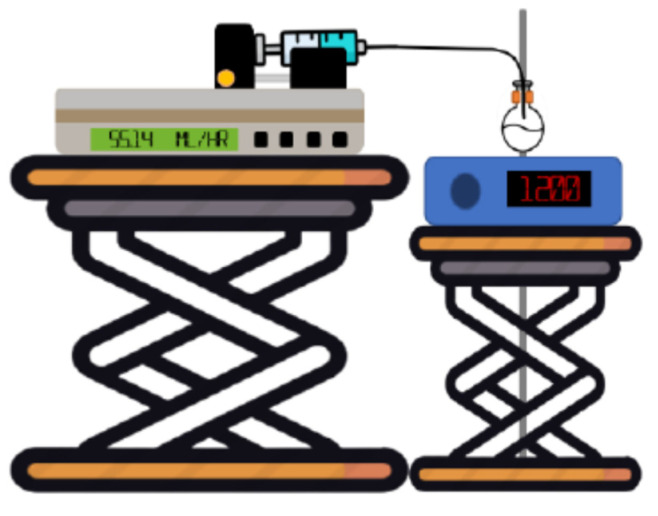
Drawing of the setup used, constructed from a Fisherbrand™ KDS100 Legacy syringe pump and 2 mL plastic syringes with Sterican (0.80 mm × 120 mm) hypodermic needles for injection into a 10 mL glass round-bottom flask placed over a Heidolph MR Hei-Tec magnetic stirrer.

The set up used for these experiments is shown in Scheme 2. This set-up was maintained constant during the experiments. It consisted of a syringe pump to control the addition rate and a stirring plate on which the stirring speed was displayed. The nanoprecipitations were performed in a 10 mL round-bottomed flask containing a 7 mm × 15 mm olive-shaped PTFE magnetic stirring bar.

The hydrodynamic diameters (Dh) and dispersity (PDI) of the prepared NPs were measured by Dynamic Light Scattering (DLS) with a Zetasizer Nano-ZR90 (Malvern) apparatus at 25 °C using a He-Ne laser at 633 nm and a detection angle of 90°. DLS measurements were performed five times.

The PMLABe_73_-based NPs encapsulating the photothermal molecular agent were prepared as follows: 5 mg of PMLABe_73_ was solubilized in 800 μL of THF and 200 μL of a solution of Ni8C12 in THF at a concentration of 2.5 mg/mL was then added so that the amount of complex was 10% of the polymer mass (10%/polymer), i.e., 0.5 mg. This solution (polymer + complex in THF) was then rapidly added to 2 mL of ultrapure water under vigorous stirring. The mixture was stirred at room temperature for 10 min, then the THF was evaporated under reduced pressure (rotary evaporator). The final volume was adjusted to 2 mL by the addition of ultrapure water. The number of dithiolene complexes effectively encapsulated into the PMLABe_73_-based NPs was determined by UV-vis-NIR titration. After nanoprecipitation of the PMLABe_73_/Ni8C12 mixture, the water solution was centrifuged and the supernatant was collected and evaporated. The residue was dissolved in THF and properly diluted to determine the exact amount of Ni8C12 encapsulated in the whole preparation. For this purpose, the molar absorptivity (ε) of Ni8C12 in THF was preliminarily determined (ε = 30,200 M^−1^·cm^−1^). The UV-Vis-NIR absorption spectra in solution were recorded on a Shimadzu UV3600 Plus spectrophotometer. The samples were placed in 1 cm path length quartz cuvettes.

Transmission electron microscopy (TEM) images were recorded using a Jeol 2100 microscope equipped with a Glatan Orius 200D camera using a 200 KeV accelerating voltage on the THEMIS platform (ISCR–Rennes). Each sample was deposited on a Formvar-carbon film-coated 300-mesh copper grid. After 6 min, the excess sample was removed and a staining was realized with phosphotungstic acid (1 volumic percent).

For the photothermal studies, 2 mL of NP suspension was irradiated through a glass cuvette with an 880 nm-wavelength semiconductor laser (Changchun New Industries Optoelectronics Tech. CO. LTD., Changchun, China) for 17 min. The power intensity of the laser could be adjusted externally (0–10 W). The output power was independently calibrated using an optical power meter. A thermocouple with an accuracy of ±0.1 °C connected to a STANDARD ST-8891E Moineau instruments thermometer was inserted into the solution. The thermocouple was inserted at such a position that direct irradiation by the laser was avoided. The temperature was measured every 1 s.

## 4. Conclusions

These investigations showed that the nanoprecipitation is a robust technique to prepare well-defined PMLABe-based NPs with a mean hydrodynamic diameter between 120 and 180 nm and dispersity between 0.07 and 0.17. The hydrodynamic diameter and the dispersity were weakly affected by the stirring speed and the addition rate but appeared to be more sensitive to the initial concentration of PMLABe in the organic solvent and the organic solvent:water ratio, even if the effects remained moderate. The initial conditions used (PMLABe_73_ solubilized into THF at a concentration of 5.01 mg/mL added into water using a THF:water ratio of 1:2 with an addition rate of 55.14 mL/h and a stirring speed of 1200 rpm) are well optimized conditions to obtain PMLABe NPs with a hydrodynamic diameter of 160 nm and a dispersity of 0.11. The present work has also revealed that hydrophobic molecular agents can be efficiently incorporated into such PMLABe-based NPs up to 50 wt% with high encapsulation efficiency, and that the obtained suspensions are highly stable over time under 4 °C storage conditions. The prepared PMLABe-based NPs encapsulating a molecular photothermal agent displayed good photothermal properties under NIR irradiation. Strong temperature increases could, in fact, be generated without disintegration of the NPs under laser irradiation. Such photothermal NPs constituted by biodegradable polymers are stable and can be considered efficient photothermal agents; they may be of great interest as contrast agents for photoacoustic bioimaging and as photothermal agents for therapy in biological media under NIR laser irradiation. Future studies will be devoted to the study of PMLABe-based copolymers encapsulating other types of metal-bis(ditholene) complexes for potential applications in nanomedicine.

## Data Availability

The data presented in this study are available on request from the corresponding authors.

## Supplementary Materials

The following are available online. Figure S1: FT-IR spectrum of A). MLABe, and B). PMLABe_73_; Figure S2: ^1^H NMR spectrum of PMLABe_73_ in CD_3_COCD_3_; Figure S3: SEC of PMLABe_73_ (THF, 40°C, 1mL/min, Polystyrene standards); Figure S4: Absorption spectra of a suspensions of PMLABe NPs incorporating 10 wt% of Ni8C12 complexes in water (C_pol_ = 300 µg·mL^−1^; C_Ni8C12_ = 30 µg·mL^−1^). Details on the encapsulation efficiency measurements.
