# Supplementary material for: Nanoprecipitation of Biocompatible Poly(malic acid) Derivative, Its Ability to Encapsulate a Molecular Photothermal Agent and Photothermal Properties of the Resulting Nanoparticles
Source: Molecules. 2021 Dec 20;26(24):7703. doi: 10.3390/molecules26247703 (PMC8703538; doi:10.3390/molecules26247703)
Supplement: Supplementary file 1 [file molecules-26-07703-s001.zip › molecules-1494248-supplementary.pdf]

Supporting information

Nanoprecipitation of biocompatible poly(malic acid) derivative, its ability to encapsulate a molecular photothermal agent and photothermal properties of the resulting nanoparticles.

Marian Gabriela Vargas Guerrero, Jean-Baptiste Pluta, Nathalie Bellec, Sandrine Cammas-Marion, Franck Camerel

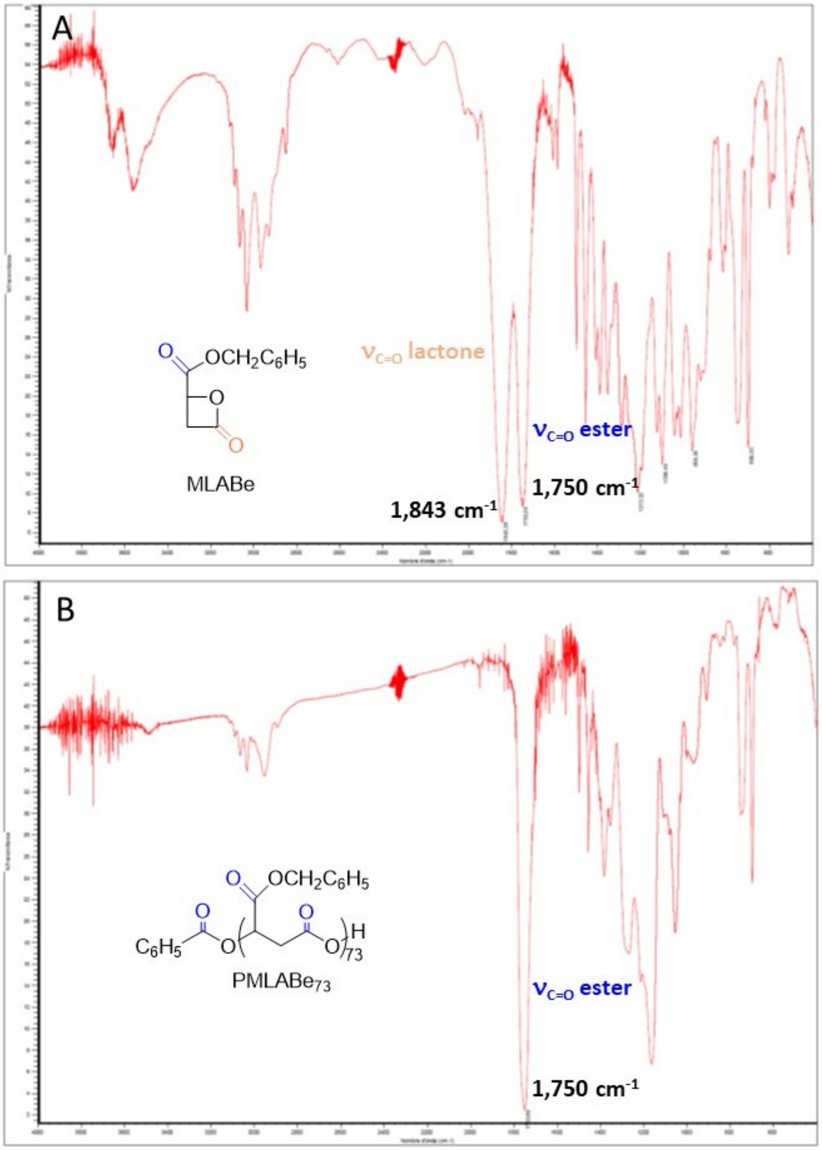

Figure S1. FT-IR spectrum of A). MLABe, and B). $PMLABe_{73}$.

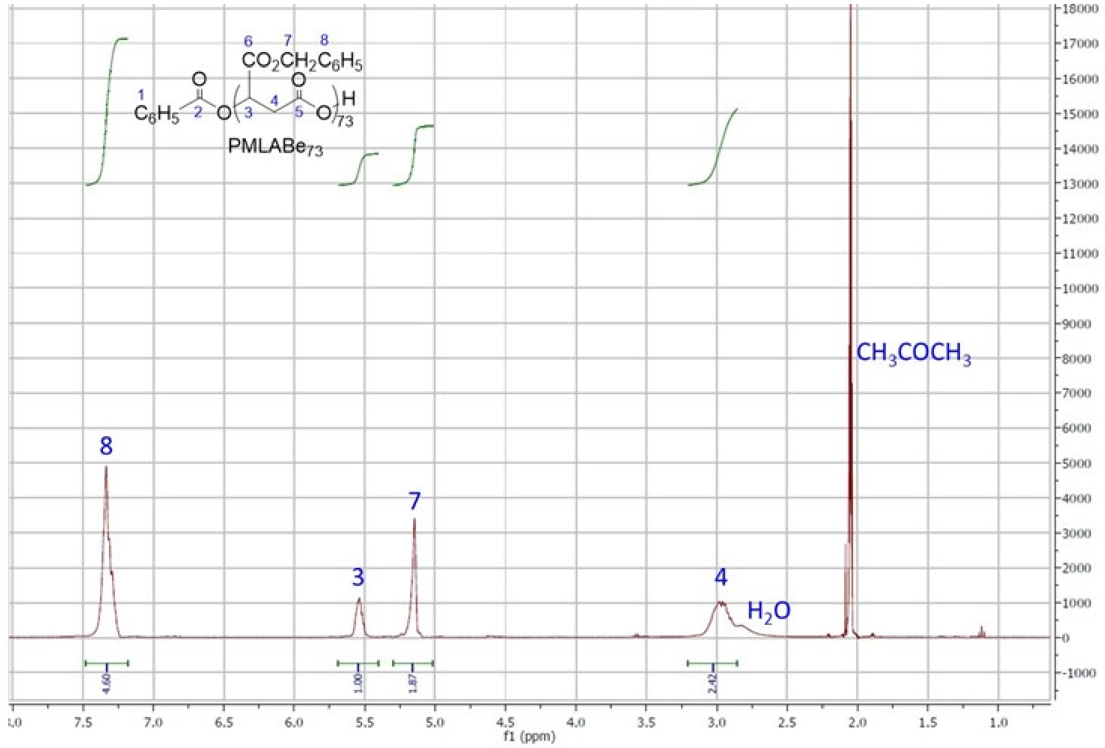

Figure S2. ^{1}H NMR spectrum of PMLABe73 in CD3COCD3.

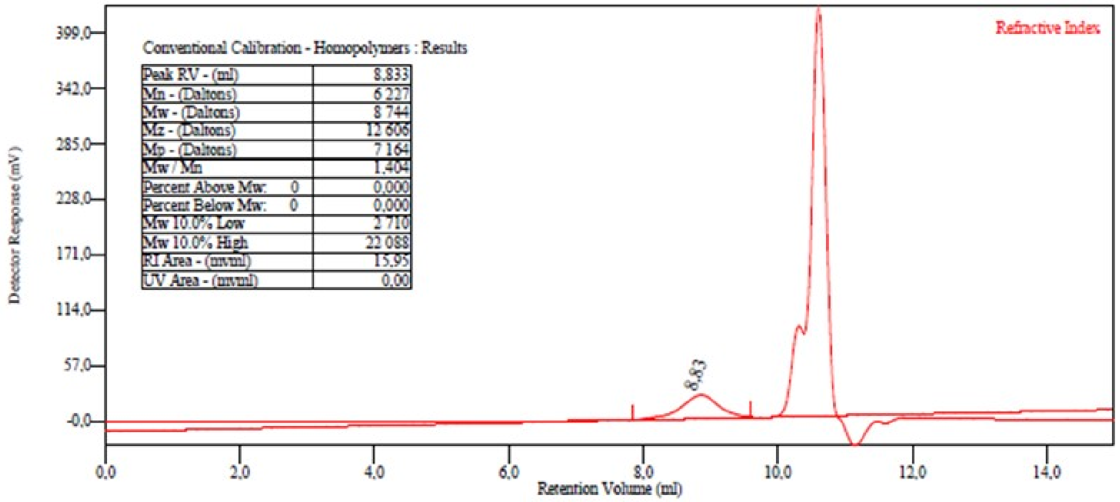

Figure S3. SEC of PMLABe73 (THF, 40°C, 1mL/min, Polystyrene standards).

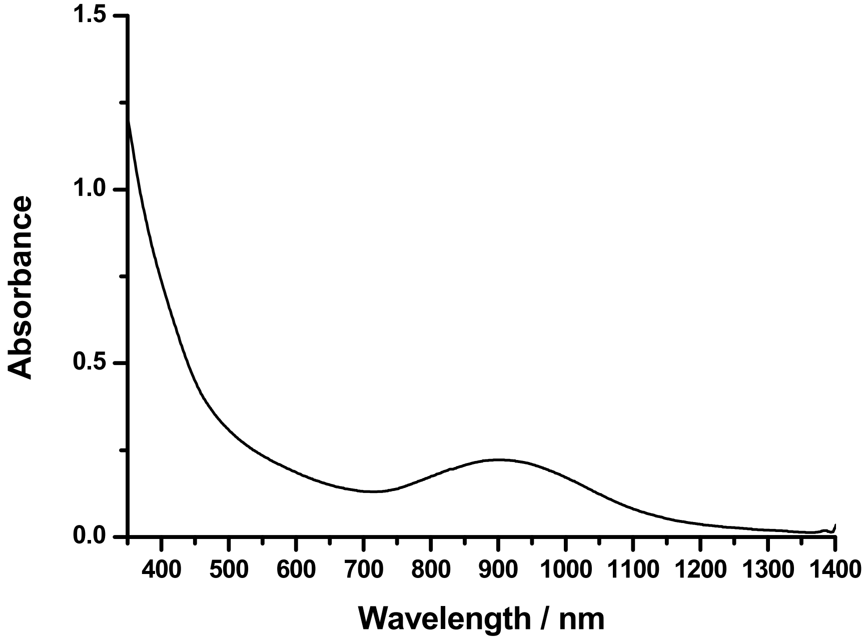

Figure S4. Absorption spectra of a suspensions of PMLABe NPs incorporating 10 wt% of Ni8C12 complexes in water (C_{pol} = 300 µg.mL^{-1}; C_{Ni8C12} = 30 µg.mL^{-1}).

The percentage of Ni8C12 encapsulation inside PMLABe nanoparticles (E.E. %) was determined by UV-Vis spectroscopy by diluting 200 µL of the nanoparticle suspensions in 2.8 mL of purified THF. In order to quantify the mass of Ni8C12 using THF as a solvent by UV-Vis spectroscopy, it was necessary to first determine the molar absorptivity (ε) of Ni8C12 in THF. For this purpose, three solutions of Ni8C12 in THF (C = 2 x 10^{-5} mol.L^{-1}) were prepared and the average ε (ε = 30200 M^{-1}.cm^{-1}) was calculated using the Beer-Lambert law. Knowing the mass of Ni8C12 in 200 µL of the nanoparticle suspension, the encapsulation efficiency was then calculated as the ratio of the mass of Ni8C12 actually encapsulated in the nanoparticles to the mass of Ni8C12 initially introduced in the encapsulation experiments (see equation 1).

E.E. % = (Weight of Ni8C12 effectively encapsulated / Weight of Ni8C12 initially introduced)x100 (1)
